# Incidence and Risk Factors for Severe Bacterial Infections in People Living with HIV. ANRS CO3 Aquitaine Cohort, 2000–2012

**DOI:** 10.1371/journal.pone.0152970

**Published:** 2016-04-06

**Authors:** Amandine Collin, Fabien Le Marec, Marie-Anne Vandenhende, Estibaliz Lazaro, Pierre Duffau, Charles Cazanave, Yann Gérard, François Dabis, Mathias Bruyand, Fabrice Bonnet, ANRS CO3 Aquitaine Cohort Study Group

**Affiliations:** 1 Centre Hospitalier Universitaire (CHU) Bordeaux, Coordination régionale de la lutte contre l’infection à VIH (COREVIH), Bordeaux, France; 2 CHU Bordeaux, Services de médecine interne et maladies infectieuses, Bordeaux, France; 3 Université de Bordeaux, ISPED, Centre INSERM U897- Epidémiologie-Biostatistique Rue Léo Saignat, Bordeaux, France; 4 INSERM U897, Centre Inserm Epidémiologie et Biostatistique, Université de Bordeaux, Rue Léo Saignat, Bordeaux, France; 5 CHU Bordeaux, Service de Médecine Interne et Maladies Infectieuses, Pessac, France; 6 CHU Bordeaux, Fédération de maladies infectieuses et tropicales, Bordeaux, France; 7 CH de Dax, Service de Maladies Infectieuses, Dax, France; FIOCRUZ, BRAZIL

## Abstract

Severe non-AIDS bacterial infections (SBI) are the leading cause of hospital admissions among people living with HIV (PLHIV) in industrialized countries. We aimed to estimate the incidence of SBI and their risk factors in a large prospective cohort of PLHIV patients over a 13-year period in France. Patients followed up in the ANRS CO3 Aquitaine cohort between 2000 and 2012 were eligible; SBI was defined as a clinical diagnosis associated with hospitalization of ≥48 hours or death. Survival analysis was conducted to identify risk factors for SBI.Total follow-up duration was 39,256 person-years [PY] (31,370 PY on antiretroviral treatment [ART]). The incidence of SBI decreased from 26.7/1000 PY [95% CI: 22.9–30.5] over the period 2000–2002 to 11.9/1000 PY [10.1–13.8] in 2009–2012 (p <0.0001). Factors independently associated to increased risk of SBI were: plasma HIVRNA>50 copies/mL (Hazard Ratio [HR] = 5.1, 95% Confidence Interval: 4.2–6.2), CD4 count <500 cells/mm^3^ and CD4/CD8 ratio <0.8 (with a dose-response relationship for both markers), history of cancer (HR = 1.4 [1.0–1.9]), AIDS stage (HR = 1.7 [1.3–2.1]) and HCV coinfection (HR = 1.4, [1.1–1.6]). HIV-positive patients with diabetes were more prone to SBI (HR = 1.6 [0.9–2.6]). Incidence of SBI decreased over a 13-year period due to the improvement in the virological and immune status of PLHIV on ART. Risk factors for SBI include low CD4 count and detectable HIV RNA, but also CD4/CD8 ratio, HCV coinfection, history of cancer and diabetes, comorbid conditions that have been frequent among PLHIV in recent years.

## Introduction

Mortality and incidence of AIDS-defining infections have dramatically decreased since the late 90’s with the advent of antiretroviral therapy (ART) [1,2]. Conversely, the declining trend in incidence of non-AIDS infections has been less pronounced. In a national study conducted in France in 2010 on causes of death, nine per cent of HIV-infected patients died from non-AIDS infections (versus 4% in 2005 and 7% in 2000 in comparable surveys) [3]. In previous studies conducted in the ANRS CO3 Aquitaine Cohort, south-western France, we have shown that non-AIDS infections accounted for more than 25% of hospitalizations in people living with HIV (PLHIV), representing the leading cause of severe morbidity in this population. Bacterial infections were the most frequent, representing 15% of causes of hospitalizations [4,5]. A recent meta-analysis has shown that bacterial infections were the second cause of hospital admission worldwide after AIDS-related illnesses [6].

Despite the improvement in the overall immune status of PLHIV on ART, community-acquired infections are more common in PLHIV than in HIV-negative individuals [7]. Observational studies have shown a decrease in hospitalizations for pneumonia since the introduction of ART, but the risk of pneumonia remains 6–8 times higher among PLHIV compared to other people living in comparable settings [8,9]. Higher risks of bacterial meningitis and pneumococcal infections have also been reported among HIV-positive individuals [10,11]. Immunosuppression, even moderate, is associated with an increased risk of bacterial, viral and fungal infections [12,13].

The impact of bacterial/viral infections on the prognosis of HIV-positive individuals remains unclear, as chronic viral infections and digestive bacterial translocation may indeed lead to chronic activation of the immune status. This activation status is associated with comorbidities in PLHIV, such as early atherosclerosis, cancers, cognitive disorders, osteoporosis [13–15]. Previous studies regarding the incidence and risk factors of severe bacterial infections (SBI) have been conducted on small cohorts, during short follow-up periods and generally before the large-scale use of ART. Moreover they have largely focused on one type of infection only (pneumonia or bacteremia) (2,7–11).

The aim of the present study was to describe the incidence and risk factors of the full scope of non-AIDS SBI in a large prospective cohort of PLHIV over a 13-year period during which ART was commonly used.

## Patients and Methods

### Patients: the ANRS CO3 Aquitaine Cohort

#### Ethics Statement

All patients included in this study provided written informed consent. The study protocol was approved by the Ethics committee of Bordeaux University Hospital (Comité de protection des personnes).

#### The ANRS CO3 Aquitaine Cohort

The ANRS CO3 Aquitaine Cohort is a prospective hospital-based cohort of HIV-1-positive patients in South-Western France. This cohort was initiated in 1987 at the Bordeaux University hospital and in four other public hospitals by the Groupe d’Epidémiologie Clinique du Sida en Aquitaine (GECSA). All adult in- or out-patients of the participating hospital wards who have an HIV-1 infection confirmed by Western blot testing, at least one follow-up visit after enrollment or a documented date of death and who provide an informed consent are eligible for inclusion.

A standardized questionnaire captures the following data at each clinic visit or hospitalization: age, gender, HIV-transmission category, clinical events since last medical contact (HIV-related or not), HIV-RNA, T-CD4 lymphocyte count, haemoglobin, hepatitis B and C serological status, ART, prophylaxis and other drugs. All events are coded according to the International Classification of Diseases 10^th^ revision (ICD10).

As of December 31^st^, 2012, 8,682 patients had been included in the Aquitaine Cohort and 3,360 of them had at least one follow-up visit recorded in 2012 [16].

### Data collection

We included in the present study all patients registered in the ANRS CO3 Aquitaine cohort and who had at least two follow-up visits in the period from January 1^st^, 2000 and December 31^st^, 2012 and for whom at least one CD4 measure was available.

A SBI was defined as a clinical diagnosis associated with hospitalization ≥48 hours or death, according to the ICD10, between 2000 and 2012.

For the purpose of the study, a specific algorithm for classifying non-AIDS infections was built from French and international guidelines [17]. More than 80% of initial diagnoses of “severe infectious events” recorded in the database were systematically reviewed by a medical doctor, who examined each patient file and categorised events as definite, probable, or possible SBI, or discarded them as a “non infectious” events. Briefly, a clinical event was considered definite if it met clinical, imaging and bacteriological criteria and had a favourable outcome with specific treatment. It was considered probable when clinical, imaging and outcomes data were concordant but there was no microbiological proof of infection. It was considered possible when only two criteria were met. Otherwise, initial diagnoses were reclassified as “non infectious event”. Six percent of the initial diagnoses of “severe bacterial infections” were reclassified as “non infectious event” and thus, were not included in the present analysis. Twenty percent of the events initially diagnosed as “severe bacterial infections” were not reviewed, mostly because the medical file of the patient was not available (deceased patients, SBI diagnosis outside public hospital, missing file). However, they were still included in the analyses, as most of the 80% of diagnoses reviewed were confirmed as correct diagnoses.

For the present analysis, we considered only the first event recorded during the study period.

Patients with a severe infection diagnosed before the beginning of the study period were excluded from the analysis.

### Variables of interest

Among the variables considered in the present analysis, age, gender, route of HIV transmission, AIDS stage, and history of cancer were measured at baseline. Hepatitis B infection (positive HBs antigen or DNA), hepatitis C infection (positive HCV antibody or RNA), and smoking status were obtained at the first measure after baseline if not available at baseline.

Other variables were considered as time-updated. Diabetes was defined by at least two fasting glycemia >7 mmol/L during follow-up, any use of antidiabetic drug or by the corresponding ICD10 diagnosis. Primary and secondary prophylactic antibiotics (trimethoprin-sulfamethoxazole, pyrimethamin, sulfadiazine and dapsone that were given according French recommendations), creatinine clearance (calculated with the simplified Modification of Diet in Renal Disease [MDRD] formula), CD4 count, CD4/CD8 ratio, plasma HIV RNA, ART use (defined by having started ART at any time), and type of ART combination (at least one protease inhibitor [PI] boosted or not, at least one non-nucleoside reverse transcriptase inhibitor [NNRTI], PI and NNRTI naive associations, and others) were considered with multiple changes.

### Data analysis

Demographic, clinical and immuno-virological characteristics including age, gender, HIV transmission group, AIDS stage, CD4 count, CD4/CD8 ratio, HIV-1 RNA level, use and type of ART, hepatitis B and C status, diabetes mellitus, and tobacco use were described in the group of patients with a SBI event with a focus on those with one of the three most frequent infections: pneumonia, bloodstream, and urinary tract infections.

Yearly incidence rates of infection were calculated by dividing the number of first infection events by the total number of patients actively followed each year. Each patient contributed to multiple years of observations, one for each calendar year. Patients could enroll in the cohort at any time preceding or during the study period, and thus the number of person-years was not constant across patients or years. If a patient was enrolled in a given year, the number of days prior to enrolment was excluded. If a patient died in a given year, the follow-up was censored on the date of death. Poisson regression was used to test for an effect of calendar period (2000–2002; 2003–2005; 2006–2008; 2019–2012) on incidence of SBI.

Kaplan-Meier method was used to calculate the probability of SBI occurrence. Analyses of the associations between potential determinants and SBI between 2000 and 2012 were performed using a Cox proportional hazards regression with time-updated covariates, censoring for deaths, lost-to-follow-up (LTFU) or end of study. Patients without any visit in the two years before 31st December 2012, while being alive and without SBI at the last visit, were considered LTFU and censored at their last visit date. Hazard ratios (HR) and 95% Confidence Intervals (CI) were calculated. Variables with p-value less than 0.25 in univariable analyses were included in a full model. No variables were forced at this stage. Final models were obtained using a backward stepwise modelling procedure. P<0.05 was considered as statistically significant. Analyses were performed using SAS 9.3 (SAS Institute, Inc., Cary, NC).

A sensitivity analysis was performed using CD4/CD8 ratio strata instead of CD4 cell count strata. A sub-analysis regarding risk factors for pneumonia was also performed; patients with another infection were censored at the time of SBI in this last model.

## Results

### Descriptive of the study sample

Between 2000 and 2012, 5,354 patients participated in the cohort; 2,512 (47%) of them were included before 2000. The median follow-up duration was 7.4 years [interquartile range (IQR): 3.2–12.3] for a total follow-up of 39,256 person-years (PY) including 31,370 PY on ART.

The number of SBI was 939 in 658 patients over the period 2000–2012. After validation, 94% of these events were classified as definite, probable or possible. LTFU rate was 27.6/1,000 PY [95% CI: 26.0–29.3] over the period 2000–2012.

**Fig 1:**
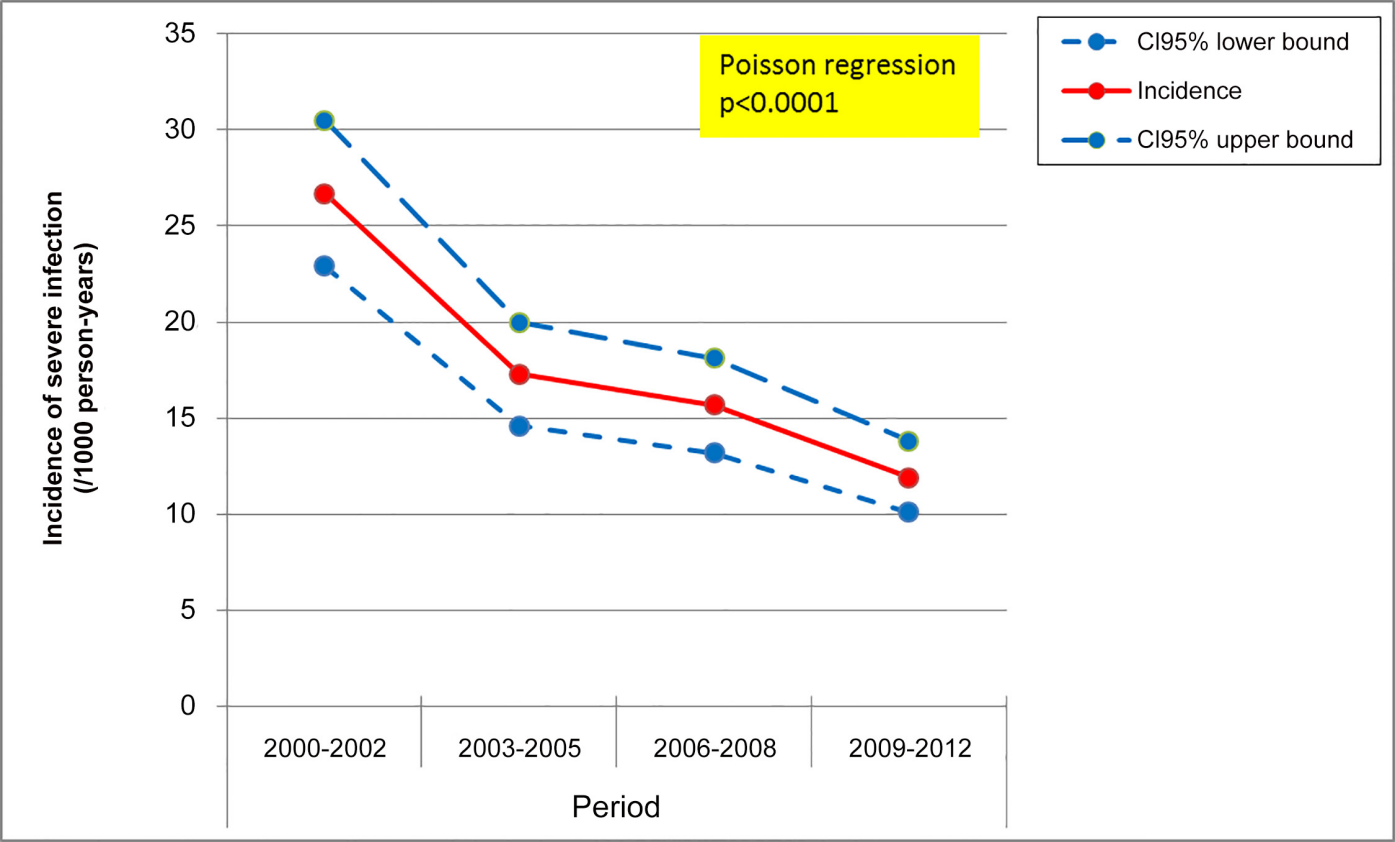
Incidence of severe bacterial infection by period. ANRS CO3 Aquitaine Cohort, 2000–2012.

The overall incidence of a first episode of SBI was 16.8/1,000 PY [95% CI: 15.5–18.0]. The incidence decreased over time, from 26.7/1,000 PY [95% CI: 22.9–30.5] in 2000–2002 to 17.3/1,000 PY [95% CI: 14.6–20.0] in 2003–2005, 15.7/1,000 PY [95% CI: 13.2–18.1] in 2006–2008 and 11.9/1,000 PY [95% CI: 10.1–13.8] in the last period 2009–2012 (Fig 1). This decreasing trend was highly significant (Poisson regression, p<0.0001).

**Fig 2:**
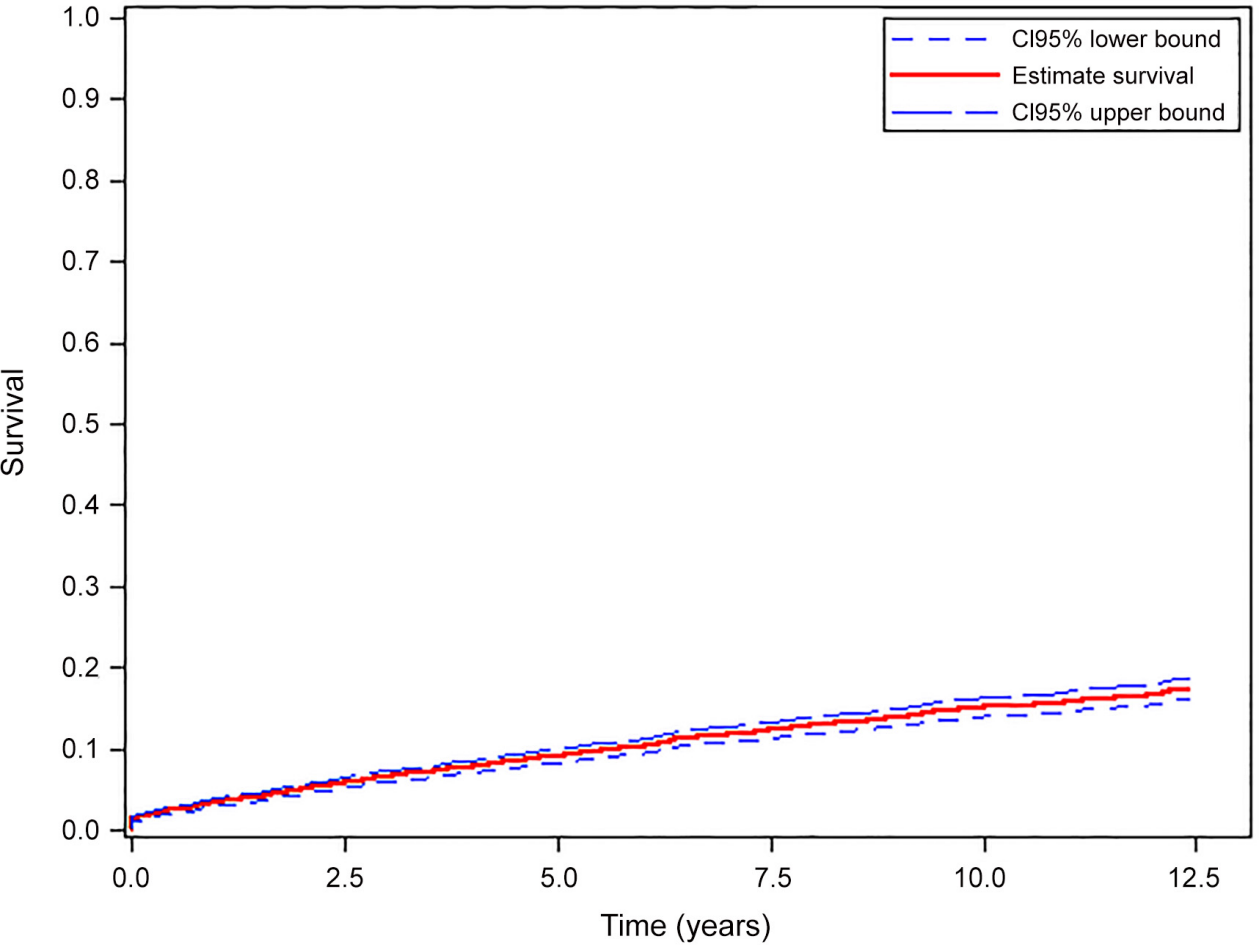
Probability of occurrence of a first episode of serious bacterial infection during follow-up. ANRS CO3 Aquitaine Cohort 2000–2012.

Fig 2 shows the probability of occurrence of a first episode of SBI during follow-up. This probability increased steadily over time to reach 9.15% [95% CI: 9.13–9.16] at five years and 15.08% [95% CI: 15.06–15.10] at 10 years.

Bacterial lung infections were the most common type of SBI with 286 cases recorded (43.5%), followed by bacteremia and urinary tract infections that accounted for 13.5% each, skin infections (11%) and digestive infections (6%). The other types of infections represented all together less than 13% of the total number of SBI.

Baseline characteristics of patients with SBI, pneumonia, bacteremia and urinary tract infections are described in Table 1 together with characteristics of cohort participants without bacterial infection.

**Table 1:** Patient characteristics at entry into the study (date of origin) by type of infections. ANRS CO3 Aquitaine cohort, 2000–2012.

		Pneumoniae (n = 286)		Bloodstream infections (n = 89)		Urinary infections (n = 88)		Severe Bacterial Infections (SBI) (n = 658)		No SBI (n = 4,696)	
**Age (**median [IQR], years)		39.6	[35.5–47.1]	40.4	[36.1–46.3]	41.6	[37.2–51.8]	39.1	[35.3–46.5]	38.9	[33.3–45.6]
**Male** (n, %)		212	(74.1)	67	(75.3)	57	(64.8)	481	(73.1)	3403	(72.5)
**Route of HIV transmission** (n, %)											
	MSM	100	(35.1)	30	(33.7)	26	(29.5)	219	(33.3)	1914	(40.8)
	Heterosexual	81	(28.4)	19	(21.3)	35	(39.8)	195	(29.7)	1646	(35.1)
	IDU	76	(26.7)	28	(31.5)	18	(20.5)	177	(26.9)	752	(16.0)
	Others	28	(9.8)	12	(13.5)	9	(10.2)	66	(10.0)	384	(8.2)
**Smoking** (n, %) ^a^		171	(76.7)	59	(77.6)	46	(73.0)	379	(75.2)	2493	(67.7)
**AIDS stage** (n, %)		90	(31.5)	35	(39.3)	25	(28.4)	210	(31.9)	746	(15.9)
**CD4 count**(median [IQR], c/mm^3^)		349	[204–540]	313	[124–501]	327	[164–513]	348	[173–538]	422	[262–600]
**CD4 nadir** (median [IQR], c/mm^3^)		195	[93–349]	150	[57–297]	194	[80–369]	195	[86–352]	297	[156–467]
**Ratio CD4/CD8** (median [IQR])		0.4	[0.2–0.6]	0.3	[0.2–0.6]	0.4	[0.2–0.6]	0·38	[0.21–0.59]	0.46	[0.28–0.72]
**HIV-RNA ≤50 copies/ml** (n, %)		59	(21.9)	17	(20.0)	20	(23.5)	143	(22.8)	1315	(29.3)
**HBV coinfection** (n, %) ^a^		27	(10.4)	12	(14.5)	3	(4.1)	57	(9.8)	401	(9.3)
**HCV coinfection** (n, %) ^a^		110	(41.4)	36	(43.4)	27	(36.5)	244	(41.0)	1076	(24.4)
**History of cancer** (n, %)		31	(10.8)	12	(13.5)	7	(8.0)	76	(11.6)	235	(5.0)
**Diabetes** (n, %) ^a^		24	(8.4)	9	(10.1)	15	(17.0)	61	(9.3)	371	(7.9)
**ART combinations** (n, %)											
	At least 1 PI or PI/r	95	(33.2)	34	(38.2)	35	(39.8)	228	(34.7)	1485	(31.6)
	At least 1 NNRTI	73	(25.5)	15	(16.9)	22	(25.0)	149	(22.6)	1044	(22.2)
	PI + NNRTI	12	(4.2)	8	(9.0)	2	(2.3)	34	(5.2)	131	(2.8)
	Others	38	(13.3)	17	(19.1)	11	(12.5)	90	(13.7)	547	(11.6)
	No treatment	64	(22.4)	15	(16.9)	18	(20.5)	150	(22.8)	1293	(27.5)
	Naïve	4	(1.4)	0	(0.0)	0	(0.0)	7	(1.1)	196	(4.2)
**TMP-SMT prevention** (n, %)		47	(16.4)	13	(14.6)	18	(20.5)	101	(15.3)	513	(10.9)
**Creatinin Clairance <90ml/mn (MDRD)** (n, %)		13	(26.0)	5	(27.8)	8	(30.8)	44	(37.3)	407	(28.5)

Legend

^a^ variables measured at the exit date; ART: Antiretroviral therapy; IDU; intravenous drug user; MSM: men who have sex with men; NNRTI: non-nucleoside reverse transcriptase inhibitor; PI: protease inhibitor.

### Determinants of SBI

In adjusted analysis, SBI occurrence was associated with AIDS stage (HR = 1.69, 95% CI: [1.33–2.14]) as well as with CDC B stage (HR = 1.45 [1.18–1.79]), HCV coinfection (HR = 1.37 [1.15–1.63]), history of cancer (HR = 1.37 [1.01–1.86]); the association with diabetes was just over the limits of statistical significance (HR = 1.55 [0.94–2.56]) (Table 2). HIV-RNA >50 copies / mL was strongly associated with an increased risk of SBI (HR = 5.08 [4.18–6.17]). The risk of SBI significantly increased below 500 CD4/mm^3^, with a higher risk for patients with CD4<200/mm^3^ and a clear inverse dose-response relationship. Interestingly also, a CD4/CD8 ratio <0·8 was also independently associated with an increased risk of SBI with again a clear inverse dose-response relationship. Route of transmission, smoking habits and antibiotics prophylaxis were not associated with the risk of SBI after adjustment.

**Table 2:** Risk factors for severe bacterial infections in HIV-infected patients, CO3 Aquitaine Cohort, 2000–2012, N = 5354 patients and 658 cases of SBI. (Cox proportional hazards regression).

Variable		Univariate analysis					Adjusted analysis(CD4+ count strata)N = 4,982^a^(Number of SBI = 616)			Adjusted analysis (CD4/CD8 ratio strata) N = 4,982 ^a^ (Number of SBI = 616)		
		N	Missing data	HR	95% CI	p	HR	95% CI	p	HR	95% CI	p
**Gender** (male vs female)		5350	4	0.95	[0.80–1.13]	0.5537						
**Route of HIV transmission**		5353	1			<0.0001						
	Heterosexual (ref)			1.00								
	Homosexual			1.05	[0.86–1.27]							
	IDU			1.85	[1.52–2.25]							
	Other			1.37	[1.04–1.81]							
**AIDS stage**		5354	0			<0.0001			<0.0001			
	A (ref)			1.00			1.00			1.00		<0.0001
	B			1.88	[1.56–2.28]		1.45	[1.18–1.79]		1.51	[1.23–1.86]	
	C			3.00	[2.51–3.59]		1.69	[1.33–2.14]		1.80	[1.42–2.28]	
**Coinfection HBV** (yes vs no)		4901	453	1.04	[0.79–1.37]	0.7626						
**Coinfection HCV** (yes vs no)		5001	353	1.86	[1.58–2.20]	<0.0001	1.37	[1.15–1.63]	0.0005	1.42	[1.19–1.69]	0.0001
**Smoking** (yes vs no)		4187	1167	1.26	[1.03–1.54]	0.0245						
**Cancer** (yes vs no)		5354	0	2.34	[1.85–2.98]	<0.0001	1.37	[1.01–1.86]	0.0431	1.37	[1.01–1.86]	0.0453
**CD4+ count strata** ^b^		5338	16			<0.0001			<0.0001			
	≥500 (ref)			1.00			1.00					
	[350–500[			1.72	[1.33–2.21]		1.41	[1.07–1.85]				
	[200–350[			4.27	[3.41–5.35]		2.72	[2.12–3.48]				
	[50–200[			7.87	[6.22–9.95]		3.75	[2.87–4.89]				
	<50			12.60	[9.42–16.86]		3.44	[2.42–4.87]				
**CD4/CD8 ratio strata** ^b^		5312	42			<0.0001						<0.0001
	≥1.0 (ref)			1.00						1.00		
	[0.8–1.0[			1.52	[1.03–2.23]					1.27	[0.85–1.90]	
	[0.5–0.8[			2.46	[1.82–3.34]					1.93	[1.40–2.67]	
	[0.3–0.5[			4.15	[3.06–5.62]					2.18	[1.56–3.05]	
	<0.3			10.95	[8.26–14.50]					3.84	[2.77–5.32]	
**Viral load >50 copies/mL** (yes vs no) ^b^		5332	22	7.64	[6.44–9.05]	<0.0001	5.08	[4.18–6.17]	<0.0001	4.99	[4.08–6.10]	<0.0001
**Diabetes** (yes vs no) ^c^		5334	20	3.78	[3.05–4.68]	<0.0001	1.55	[0.94–2.56]	0.0871	1.57	[0.95–2.58]	0.0785
**Cotrimoxazole prophylaxis** (yes vs no) ^b^		5354	0	1.42	[0.92–2.20]	0.1139						
**ART** ^b^		5354				0.0070						
	No treatment (ref)			1.00								
	PI-based			1.28	[1.03–1.59]							
	NNRTI-based			1.16	[0.92–1.48]							
	PI and NNRTI			2.00	[1.36–2.96]							
	Other			1.31	[1.00–1.72]							
**ART** (treated vs no treated) ^b^		5354	0	1.28	[1.05–1.55]	0.0127						
**Creatinin clearance <90mL/min** ^b^		4650	704	1.13	[0.93–1.39]	0.2240						

Legend

^a^ Missing data for HCV coinfection or CD4+ count strata / CD4/CD4 ratio strata or viral load >50 copies/mL

^b^ Time-updated variable with multiple change

^c^ Time-updated variable with unique change. Abbreviations: ART: Antiretroviral therapy; IDU; intravenous drug user; MSM: men who have sex with men; NNRTI: non-nucleoside reverse transcriptase inhibitor; PI: protease inhibitor.

A sub-analysis was conducted regarding the risk factors for lung infections. HCV coinfection, low CD4 count, low CD4/CD8 count, HIVRNA >50 copies/mL were independently associated with an increased risk of developing severe pneumonia (data not shown).

## Discussion

In this large observational and prospective study including more than 5,000 patients and with accurate diagnostic and validation procedures, we report that the incidence of non-AIDS severe infections significantly decreased over a 13-year-period in the ART era. However, it remains higher than that of any other severe comorbidity (including AIDS events) especially in the most recent years [18].

Our study is, to our knowledge, the first to describe the incidence of non-AIDS severe bacterial infections over a long-term follow-up period, within a cohort of patients receiving good standard of care and fairly standardized across sites. Of course, the high reduction in SBI incidence we observed over time may reflect the general improvement of HIV control thanks to the increasing use of ART. Indeed, we have shown that a low CD4 count remains a strong risk factor for SBI as well as uncontrolled HIV RNA replication. If the results regarding the association between CD4 count and bacterial infections were expected, although rarely described, the independent association between HIV RNA replication with the risk of occurrence of SBI and pneumonia remains difficult to explain. This finding has already been identified in few cohorts [17], suggesting that HIV replication may induce a state of immunosuppression, regardless of CD4 count, through a qualitative restriction of the immune response to bacterial agents. Indeed, HIV infection seems able to affect many components of the immune response such as epithelial barriers, macrophage phagocytosis, NK cell anergy, neutrophil-mediated host defense, humoral immunity [19]. This finding further confirms the clinical benefits of universal treatment of HIV infection regardless of CD4 count, a recommendation that was adopted in France in 2013, i.e. after the end of the study period [20].

Interestingly, we have shown for the first time that a CD4/CD8 ratio <0.8 was associated with an increased risk of bacterial infection. A low CD4/CD8 ratio has already been reported to be associated with certain chronic comorbidities (cardiovascular events, renal failure, Hodgkin diseases, non-AIDS cancers) and with AIDS and non-AIDS mortality [21,22]. We are now showing that it is also associated with acute and frequent morbidity events such as bacterial infections. Thus, at a time when more than 85% of patients on ART in France reach the goal of undetectable HIV RNA and more than half reach the goal of 500 CD4/mm^3^, using the CD4/CD8 ratio level may help clinicians define a subgroup of patients with phenotype of T-cell activation and senescence, and with a higher risk of chronic but also acute morbid events. We identified other non-HIV determinants of SBI such as HCV coinfection, history of cancer and diabetes. The associations between HCV infection, intravenous drug use and bacterial infections, particularly pneumonia, have been described in the literature [23–26] but information is scarce among HIV-infected patients. The associations with diabetes (just above the limits of statistical significance) and with history of cancer have not been described in the context of HIV infection. In non-HIV populations, these last two conditions have been described as independent risk factors for bacterial infections [27–31].

The use of ART was not associated with a decreased risk of severe infection in our study but the fact that ART coverage was very high in our study population (> 95% in 2013 and 80% over the period 2000–2012) makes it difficult to interpret these last results.

Among non-AIDS severe infections, pneumonia, bacteremia and urinary infections were the most frequent infections associated with hospitalizations followed by skin and digestive tract infections. Pneumonia remains one of the major clinical risks in PLHIV and recurrent pneumonia has been classified as AIDS for many years. We have shown previously that, in addition to better control of HIV infection, tobacco smoking cessation was associated with a decreased risk of pneumoniae [32]. Vaccination against *Pneumococcus* and *Haemophilus* influenzae are critical preventive measures for this population but they were not systematically implemented at the time of the study and insufficiently documented to be able to take this information into account in the analysis. Vaccine efficacy in HIV population is still subject to debate and may depend on the degree of immunosuppression [33]. Should vaccine efficacy be lower than in the general population, considering our findings, it is of utmost importance to propose such vaccination to every PLHIV.

In our series of SBI, 13.5% of our patients had bloodstream infections. Information on bacterial bloodstream infections in PLHIV is scarce. Few studies have shown an increased risk of bloodstream infections among PLVIH but most of them were conducted in resource-limited settings or before the ART era [34]. However, a low CD4 count and the absence of ART seem to be the most important risk factors for bloodstream infections [7]. In addition, in our study, 13.5% of HIV-positive individuals with bloodstream infections had a previous history of cancer and 10.1% were diabetics, suggesting that not only HIV conditions but also comorbidities should be considered as risk factors for bloodstream infections.

Urinary tract infections accounted for 13.4% of severe infections in the present series. Data on these infections are also scarce in PLHIV. We have noticed that, similar to other bacterial infections, CD4 count at the time of occurrence was low and that more than 17% were also diabetics. Diabetes is highly associated with urinary tract infections in the general population [35].

Our study has several limitations. Within this real-world cohort, it is possible that not all SBI events among the HIV-positive patients followed-up were recorded in our database. However, we believe it would have been a rare event since most of the public hospitals of the region are included in the cohort network. Moreover, like other severe events requiring hospitalization, the systematic assessment of patient records would have corrected this missing event. The rate of patient LTFU was quite low in our cohort. An active tracking system of LTFU patients is implemented in the cohort and captures additional fatal events. Thus, we believe that we are probably close to exhaustive reporting of SBI in the cohort. Moreover, a clinician specialist validated all events were using a specific algorithm prior to analysis. The main limitation of the study is the lack of information regarding vaccination against flu and pneumonia. We are not able to conclude on the efficacy of vaccination in this population and this issue should be further evaluated.

## Conclusion

We have shown in this large cohort study a decreasing incidence of SBI over a 13-year follow-up period. However, SBI remain the main cause of morbidity among PLHIV and a challenge for research. Furthermore, this finding highlights the need for strong vaccination programs for PLHIV.

The main risk factors for SBI in our setting include low CD4 count, HIV RNA replication, HCV coinfection, but also a history of cancer and diabetes, comorbid conditions that have been frequent in PLHIV in recent years. Finally, regardless of the fact that subgroups of HIV-positive patients are more at risk of SBI, these results support the long-term benefits of universal and early use of ART.
